# Acral lentiginous melanoma: Basic facts, biological characteristics and research perspectives of an understudied disease

**DOI:** 10.1111/pcmr.12885

**Published:** 2020-06-17

**Authors:** Patricia Basurto‐Lozada, Christian Molina‐Aguilar, Carolina Castaneda‐Garcia, Martha Estefania Vázquez‐Cruz, Omar Isaac Garcia‐Salinas, Alethia Álvarez‐Cano, Héctor Martínez‐Said, Rodrigo Roldán‐Marín, David J. Adams, Patricia A. Possik, Carla Daniela Robles‐Espinoza

**Affiliations:** ^1^ Laboratorio Internacional de Investigación Sobre el Genoma Humano Universidad Nacional Autónoma de México Santiago de Querétaro Mexico; ^2^ Tecnologico de Monterrey School of Engineering and Sciences Centre of Bioengineering Querétaro Mexico; ^3^ Wellcome Sanger Institute Hinxton Cambridgeshire CB101SA UK; ^4^ Instituto Nacional de Cancerología (INCan) Mexico City Mexico; ^5^ Dermato‐Oncology Clinic Unidad de Medicina Experimental Facultad de Medicina Universidad Nacional Autónoma de México Mexico City Mexico; ^6^ Program of Immunology and Tumor Biology Brazilian National Cancer Institute (INCA) Rio de Janeiro Brazil

**Keywords:** acral melanoma, diagnosis, epidemiology, genomics, microenvironment

## Abstract

Acral lentiginous melanoma is a histological subtype of cutaneous melanoma that occurs in the glabrous skin of the palms, soles and the nail unit. Although in some countries, particularly in Latin America, Africa and Asia, it represents the most frequently diagnosed subtype of the disease, it only represents a small proportion of melanoma cases in European‐descent populations, which is partially why it has not been studied to the same extent as other forms of melanoma. As a result, its unique genomic drivers remain comparatively poorly explored, as well as its causes, with current evidence supporting a UV‐independent path to tumorigenesis. In this review, we discuss current knowledge of the aetiology and diagnostic criteria of acral lentiginous melanoma, as well as its epidemiological and histopathological characteristics. We also describe what is known about the genomic landscape of this disease and review the available biological models to explore potential therapeutic targets.

## 1 INTRODUCTION

Cutaneous melanoma (CM) (for this review, we will consider cutaneous melanoma as all melanomas that arise on skin) is usually classified mainly into four main histological subtypes: superficial spreading melanoma (SSM), lentigo maligna melanoma (LMM), nodular melanoma (NM) and acral lentiginous melanoma (ALM). ALM arises in acral locations, specifically in palms, soles and the nail unit. The word “lentiginous” refers to its radial growth phase, which appears before the tumour starts invading the dermis. The terms “lentiginous” and “acral” might not be used always together, and could cause some confusion even among pathologists and clinicians. Before we delve deeper into its characteristics, some definitions are in order: as ALM is the most common histopathological subtype of melanoma that arises in acral sites, some authors use acral melanoma and ALM as interchangeable terms (Darmawan et al., 2019), while other authors refer to acral melanomas as all histopathological subtypes that occur on the glabrous skin of the palms, soles or nail unit. Additionally, there are authors that include melanomas from dorsal surfaces of the hands and feet in the category of acral melanomas (Haugh et al., 2018). However, it is important to note that not all melanomas that arise on acral locations are ALMs, as they can also be of the nodular or superficial spreading subtypes, particularly on the dorsum of the hands or feet or other sun‐exposed areas of acral location.

Although ALM represents the majority of melanoma cases in some Latin American, African and Asian countries, it comprises a relatively low per cent of melanoma diagnoses in countries with primarily European‐descent inhabitants such as the United States, the United Kingdom and Australia (Ossio, Roldán‐Marín, Martínez‐Said, Adams, & Robles‐Espinoza, 2017). Because ALM incidence rates across populations appear to be similar (Wang, Zhao, & Ma, 2016), this large gap is likely accounted for by the strikingly different incidence rates of sun‐induced melanoma. All in all, this has meant that ALM remains understudied, with its predisposing factors, causal agents and mutational drivers mostly unknown. As a consequence, patients still lack effective treatment options.

Here, we start by briefly reviewing our current knowledge regarding ALM causes and risk factors, its incidence trends around the world and diagnostic strategies. We then discuss known genomic drivers, and its biological and histopathological characteristics. Finally, we review the current status of available disease models and discuss therapeutic possibilities. In each section, we have strived to give an interpretation of incomplete or conflicting data where they arise, and pinpoint areas where more evidence is needed to reach a consensus.

## 2 EPIDEMIOLOGY

The aetiology of ALM remains controversial. Although so far, no familial cases of ALM have been reported, there is scattered evidence suggesting that some genetic risk factors might exist. For instance, a large cohort study observed an increased risk of any major melanoma subtype in patients with first‐degree relatives diagnosed with ALM (Fallah et al., 2014), suggesting some shared genetic factors with other melanoma subtypes. Another study found that ALM patients had secondary cancers and a family history of cancer more often than patients with other melanoma subtypes (Nagore, Pereda, Botella‐Estrada, Requena, & Guillén, 2009). Similarly, another large study showed a positive association between the number of naevi and risk of acral melanoma (Green et al., 1999) or of rare melanoma subtypes, which included ALM (Newton‐Bishop et al., 2010). Yet, there is no clear aetiology for ALM, and due to its development on sun‐shielded locations, it is believed not to be related to UV exposure. Regardless of its biological origin, a history of trauma has frequently been proposed as an ALM trigger, since tumours develop on weight‐bearing areas of the body or sites that are highly susceptible to mechanical injury such as palms and soles. In this regard, a retrospective study on 685 Chinese acral melanoma patients found an association between prior trauma and disease development at the tumour site (Zhang et al., 2014). Additionally, other studies have indicated that the most common location where ALM arises is the foot, in accordance with this being the site under the highest mechanical stress, and have suggested that tumour location in this region overlaps with the highest pressure areas on the plantar foot (Al‐Hassani, Chang, & Peach, 2017; Jung, Kweon, Lee, Lee, & Yun, 2013; Minagawa, Omodaka, & Okuyama, 2016; Sheen et al., 2017). However, the trauma theory remains contentious, as other studies have not found this correlation; for example, a retrospective study of 122 acral melanomas from the Mayo Clinic found no significant difference in the distribution of acral melanomas on weight‐bearing and non‐weight‐bearing regions (Costello, Pittelkow, & Mangold, 2017). Additionally, analyses of the incidence of ALM in African tribes did not find differences in incidence between groups who wore shoes or were barefoot, although sample sizes were limited (Barra‐Martínez, Herrera‐González, Fernández‐Ramírez, & Torres, 2015; Rippey, Rippey, & Giraud, 1975).

Given the observations cited above, the origin of this disease may be multifactorial, characterized by an interaction between common genetic variants of small effect and certain environmental cues, such as trauma (Fallah et al., 2014; Newton‐Bishop et al., 2010; Sheen et al., 2017). Gene/environment interactions have previously been demonstrated for other melanoma subtypes, for which both genetic variants in genes such as *MC1R* and environmental exposures such as UV light are established risk factors. Usually, large‐scale case/control cohort genotyping and genome‐wide association testing are necessary to elucidate the influence of these aetiological agents; however to date, no such studies have been published for ALM.

Regarding incidence trends, ALM shows similar rates across ethnicities (Ridgeway, Hieken, Ronan, Kim, & Das Gupta, 1995; Wang et al., 2016). However, it represents contrasting proportions of melanoma cases across populations due mainly to marked differences in sun‐induced melanoma incidence, which is much more common in fair‐skinned individuals. Whereas ALM represents between 1% and 8% of the melanoma diagnoses in European‐descent individuals, this proportion is much higher in Hispanic, African‐ and Asian‐descent populations, and in some countries, it can constitute more than 50% of cases (Bradford, Goldstein, McMaster, & Tucker, 2009; Lee, Chay, Tang, Chio, & Tan, 2012). Here, we have decided to focus on studies from non‐European populations, as incidence rates in European populations have been discussed extensively by others (Bradford et al., 2009; Kuchelmeister, Schaumburg‐Lever, & Garbe, 2000; Minini, Rohrmann, Braun, Korol, & Dehler, 2017; Teramoto et al., 2018).

### 2.1 Latin America

We can hypothesize that countries in Latin America have varying proportions of ALM perhaps reflecting the amount of European, Indigenous and African ancestries in their population and therefore their distinct susceptibility to UV‐induced melanoma. However, accurate incidence estimates are difficult to obtain due to the lack of national cancer registries.

In countries with a higher European ancestry such as Argentina, more than half of melanoma cases are of the SSM subtype and ALM represents approximately 6% of diagnoses, as a study of 3,832 cases reported (Loria & González, 2013). In Brazil, an analysis of patients mostly of European descent reported that the most prevalent subtype was SSM (Vazquez et al., 2015), and another study of 3,878 patients suggested that only 13.6% of melanoma cases were located in acral sites, of which the most frequent histopathological subtype was ALM (44.3%). Interestingly, this study also reported that 56.8% of the patients diagnosed with melanoma in acral sites self‐declared as White, while the other 43.2% self‐declared as non‐White (Nunes, Quintella Mendes, & Koifman, 2018). In Chile, a study of 1,148 melanoma cases showed differences in anatomical site presentation according to site of collection (state hospitals vs. private clinics), which is a proxy for socioeconomic status (SES) (Zemelman, Valenzuela, Sazunic, & Araya, 2014). Patients of high SES showed a lower proportion of acral melanoma (~2% of all melanoma diagnoses) and a lower proportion of Amerindian ancestry (~20%) compared with patients of low SES, whose acral melanoma proportion was ~10% and who had about 40% Amerindian ancestry. An older, smaller study in the same country showed SSM as the most common subtype of the disease with 37.5% of the cases, followed by NM (31.2%) and acral melanoma (22.1%) (Cabrera et al., 1994). In Mexico and Peru, countries with a complex demographic history with European, African and Native American ancestries, ALM comprises most of the melanoma diagnoses (Coras, Morales, Yabar, & Beltran, 2013; del Carpio, 2008; Lino‐Silva et al., 2016). These differences in melanoma subtype presentation reflect the complex aetiology of the disease, among which are the existence of different ethnic groups within these populations and the disparity of healthcare access correlating with ancestry.

### 2.2 Africa

There are limited studies regarding ALM prevalence in African countries. In general, epidemiological studies have observed a higher proportion of acral melanoma in Black African populations compared with White African populations, consistent with observations in other countries around the world. For example, a study by Hudson and Krige from 1972 to 1985 in South Africa reported that melanoma on the plantar surface accounted for 71% of all CMs in Black African patients, with 54% of all CMs identified as ALM (Hudson & Krige 1995). Another study in the same country reported that 80% of CM diagnoses in Black individuals are in acral sites, and accordingly, that the majority of acral melanoma diagnoses are made in Black individuals (De Wet, Tod, Visser, Jordaan & Schneider, 2018). A more recent study, also performed in South Africa using data collected from 2008 to 2012, reported that ALM was the most common subtype observed and that all ALM cases came from the public health system. In this health system, Black Africans represented 43% of patients, similar to the ALM‐SES relationship observed in Chile by Zemelman *et al*. This study also reported that ALMs were often diagnosed at advanced stages (York et al., 2016). A study from Togo compiled 63 cases of melanoma and reported similar results, where acral lentiginous lesions were the most common (Pitché, Napo‐Koura, & Tchangai‐Walla, 2005). This was also the case in Tunisia where Naouali et al. (2017) reported ALM as the most frequent histological subtype (Naouali et al., 2017).

### 2.3 Asia

As in the case of Hispanic and African populations, the most common form of melanoma in Asian populations is ALM. A study in Singapore estimated that 50% of melanoma patients were diagnosed with this subtype (Lee et al., 2012), with others estimating that this proportion is 47% for Japanese patients (Ishihara et al., 2008), 42% for Chinese patients (Chi et al., 2011) and 58% for Taiwanese patients (Chang et al., 2004). A study of 206 cases ascertained from 2005 to 2012 in Korea found that 45.6% presented with acral melanoma (Jang, Kim, Park, et al., 2014), and a study in the United States estimated that Asian Americans were significantly more likely to be diagnosed with ALM than Non‐Hispanic Whites (Shin, Palis, Phillips, Stewart, & Perry, 2009). Similar to Hispanics and Africans, UV exposure is unlikely to be an important aetiological factor for ALM development and predisposition, and then again, trauma has been proposed to play a role in the disease, although no conclusive evidence of causation has been found (Bellew, Del Rosso, & Kim, 2009).

## 3 DIAGNOSIS

When diagnosed early, melanoma is often curable through a surgical excision of the lesion. Unfortunately, the diagnosis of early‐stage ALM remains challenging as diagnostic features are often subtle (Darmawan et al., 2019; Fernandez‐Flores & Cassarino, 2017). ALM is histologically characterized by a diffuse proliferation of large atypical melanocytes along the dermoepidermal junction with a lentiginous growth pattern with marked acanthosis and elongation of the rete ridges (Darmawan et al., 2019).

ALM lesions are often diagnosed at later stages, and it is common for them to be misdiagnosed as fungal infections, warts, diabetic foot ulcers or traumatic ulcers (Criscito & Stein, 2017). Furthermore, differential diagnosis may be complex considering other pigmented (pigmented poromas) and unpigmented and bleeding tumours (pyogenic granulomas) often seen on acral skin. Misdiagnosis has been observed to be associated with increased median tumour thickness, more advanced stage at diagnosis and lower 5‐year survival (Soon et al., 2003). Misdiagnosis is also associated with low SES, lack of prompt access to assessment by a dermatologist, lack of full body examination during regular primary care physician consultation, low interest and insufficient education regarding skin cancer in the medical school curricula and a highly demanded and saturated public healthcare system.

**FIGURE 1:**
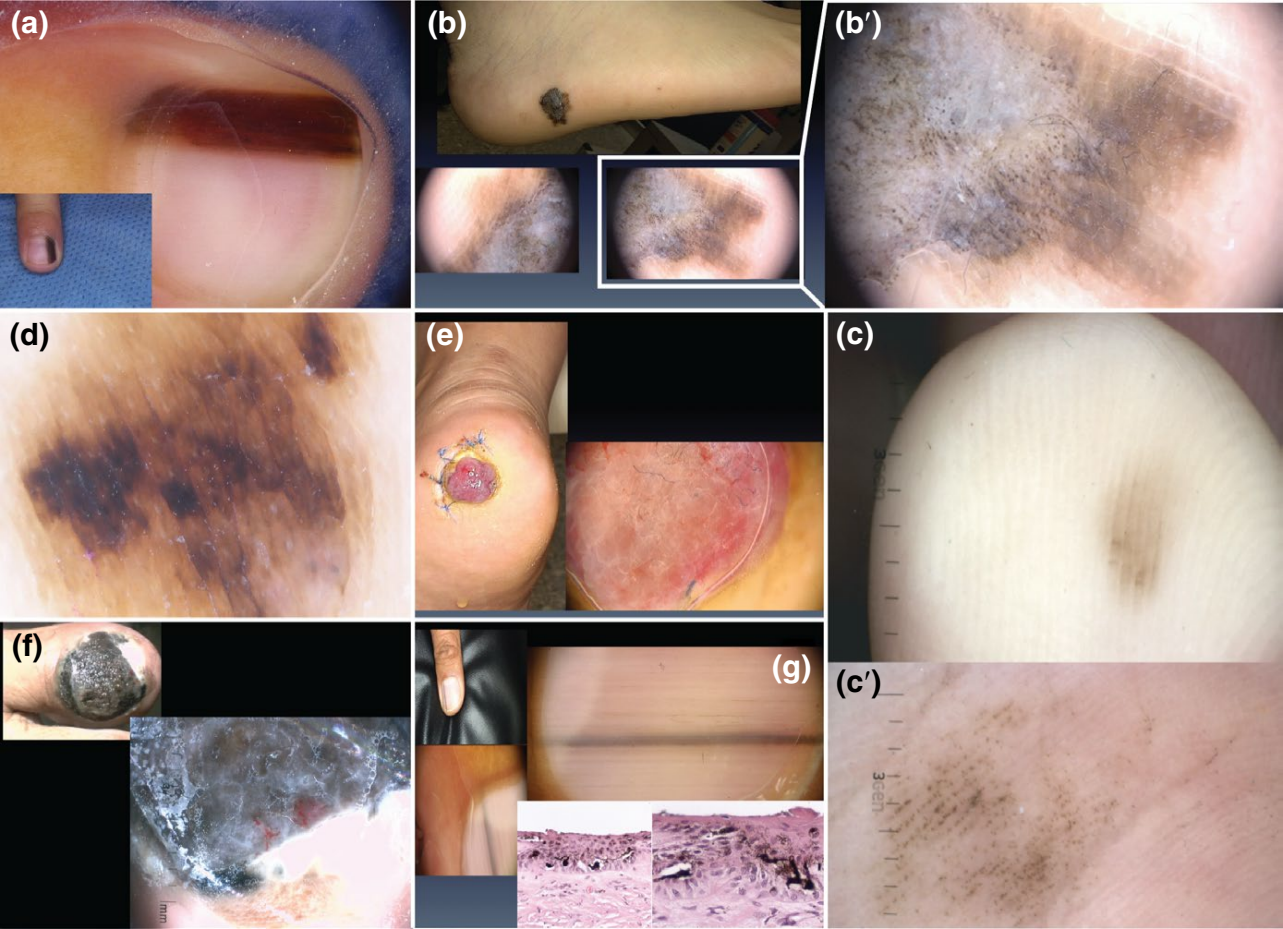
Examples of distinct acral lesions. (a) Longitudinal melanonychia in a 6‐year‐old patient with a subungual naevus. (b) Clinical appearance of an acral lentiginous melanoma (top panel), for which dermoscopy shows a parallel ridge pattern (bottom panels). (b′) Close‐up of the parallel ridge pattern typical of acral lentiginous melanoma. (c) Clinical appearance of an acral naevus showing a parallel furrow pattern. (c′) Close‐up of the parallel furrow pattern typical of acral naevi. (d) Dermoscopic appearance of a diffuse irregular pigmentation pattern in an advanced‐stage acral lentiginous melanoma. (e) Clinical and dermoscopic appearance of an amelanotic nodular melanoma on the heel. (f) Advanced‐stage nail unit melanoma. Dermoscopy is not necessary to make a diagnosis in these cases. (g) Adult patient with new onset of a pigmented lesion on the nail unit; clinically, a longitudinal melanonychia affecting the first finger. The recent onset, the affection of the first finger and the grey coloration shown by dermoscopy were important clues to suggest this was a nail unit melanoma despite the fact that there was no loss of parallelism nor affection >2/3 of the nail plate. All patients depicted here signed an informed consent that authorized the use of their photographs for research purposes

Melanoma detection depends heavily on an adequate physical examination of the skin, as well as in the experience of the medical professional who performs it. The ABCDE criterion for the assessment of cutaneous lesions has been used as a successful screening tool to help in the identification of melanoma‐prone skin lesions (Balch et al., 2009). Nevertheless, the accuracy of the ABDCE rule when diagnosing melanoma in the foot and nail unit has been questioned due to differences in tumour presentation in ALM compared with other CM subtypes (Albreski & Sloan, 2009). In light of these, an alternative acronym, CUBED, which stands for Coloured lesion, Uncertain diagnosis, Bleeding lesion on the foot or under the nail, Enlargement of a lesion and Delay in healing, was proposed for the identification of ALM lesions (Bristow & de Berker, 2010); however, this acronym lacks specific morphologic criteria (Darmawan et al., 2019). Subungual lesions also tend to be misdiagnosed due to their unusual location and presentation, especially if the healthcare provider is not familiar with this type of melanoma. For example, longitudinal melanonychia (Figure 1a), a sign of nail unit melanocyte activation (in some cases related to melanocytic hyperplasia with or without abnormal melanocyte proliferation) that gives the nail plate a brown or black colour is present in two thirds of nail melanoma cases, but can also be present in nail unit naevi or when there is trauma or onychomycosis (André & Lateur, 2006).

Given the above difficulties in diagnosis, dermoscopy is widely seen as the best method to perform a prompt diagnosis of ALM. This tool is useful for the evaluation of skin lesions because it increases the diagnostic accuracy over naked eye examination by 30% when performed by an experienced physician (Braun, Rabinovitz, Oliviero, Kopf, & Saurat, 2005). It has also been shown to improve discrimination between acral melanoma and acral naevi mainly by highlighting the accentuation of the pigmentation on the skin ridges (parallel ridge pattern or parallel furrow pattern). ALM lesions most commonly display a parallel ridge pattern (Figure 1b,b′), while acral naevi display a parallel furrow pattern (Figure 1c,c′), lattice‐like pattern or a fibrillar pattern. However, not all acral melanomas present with a parallel ridge pattern (Lallas et al., 2015), and some benign lesions such as drug‐induced acral pigmentation, subcorneal haemorrhage, and lentigines of Peutz–Jeghers syndrome and Laugier–Hunziker syndrome have been reported to also present with this pattern (Darmawan et al., 2019). Therefore, Lallas et al. (2015) suggest to consider additional features when evaluating a pigmented lesion and have proposed the “BRAAFF” checklist as a tool to help detection of acral melanomas that deviate from the parallel ridge pattern. This checklist delivers a score based on the presence of the following criteria: irregular blotch, parallel ridge pattern, asymmetry of structures, asymmetry of colours, parallel furrow pattern and fibrillar pattern. However, ALM diagnosis for patients of non‐European descent can still be challenging as benign congenital acral pigmented lesions are more common in dark‐skinned patients. These congenital acral naevi can show a highly pigmented blotch in the central area, often black‐grey or blue‐grey coloured, with dark brown projections peripherally (parallel furrow pattern most of the time).

Advanced‐stage ALM usually shows an irregular diffuse pigmentation pattern. It is characterized by the presence of multiple structureless areas of pigmentation of different shades of brown, black and grey, generally arranged asymmetrically and irrespective of the dermatoglyph architecture (Lallas et al., 2015) (Figure 1d).

Unpigmented benign or malignant tumours also exist on acral skin, their diagnosis is based on the analysis of their vascular pattern. However, diagnostic rules about vascular patterns of acral skin do not differ from those concerning other locations. Attention should be given to pigmented structures only observed by dermoscopy and invisible to the naked eye, in the diagnostic approach to unpigmented or hypopigmented acral tumours, so as not to miss amelanotic melanoma (Figure 1e). The “EFG” acronym (Elevated, Firmness and Growth) has been proposed to avoid missing amelanotic melanoma (Giacomel, Zalaudek, Mordente, Nicolino, & Argenziano, 2008).

Diagnosis of nail unit melanoma is often delayed by a poor general knowledge of its clinical characteristics, and a many‐year history of a misdiagnosed condition is common at a patient's first interrogation. Dermoscopy is often not needed in advanced cases but allows a very early diagnosis in paucisymptomatic cases characterized only by longitudinal melanonychia. Early pigmented nail unit melanoma is characterized by the presence of a brown‐grey background of the pigmentation and an irregular pattern of the longitudinal microlines, only dermoscopically visible. These lines are irregular in colour, thickness, spacing and parallelism. Occasionally, an only‐dermoscopically visible periungual pigmentation (also known as the micro‐Hutchinson sign) is seen on the proximal nail‐fold skin (Littleton, Murray, & Baratz, 2019). More advanced stages of pigmented nail unit melanoma may be dermoscopically characterized by granulation, scar‐like depigmentation, blue‐black structureless areas, blood spots, prominent periungual pigmentation, atypical vascular areas, erosion of the nail plate or ulceration of the nail bed. In these cases, clinical diagnosis is usually sufficient (Figure 1f). Affection of the first finger or toe, pigmentation of two thirds of the nail plate, presence of black‐grey pigmentation, irregularly sized and coloured band and Hutchinson and micro‐Hutchinson signs are important clues that aid in the diagnosis of nail unit melanoma (Benati et al., 2017) (Figure 1g).

Local recurrence is two to five times higher for ALM than for melanoma at other sites and can be associated with poor survival. Wide excision is the primary treatment modality. The size of the excision is challenging in ALM since a smaller margin favours minimal functional impairment, but a wider excision may be more likely to be curative. One possible explanation for the high local recurrence rate is the finding of genetically abnormal melanocytes in histologically normal epidermis adjacent to melanomas on the non‐hair‐bearing skin of the palms and soles (Bastian et al., 2000; North, Kageshita, Pinkel, LeBoit, & Bastian, 2008). These “field” cells were not histologically identified because they were not sufficiently increased in number, abnormally distributed or markedly atypical compared with normal melanocytes. Thus, field cells may be a form of occult melanoma that, if left behind, lead to local recurrence through a subtle contiguous extension of the primary melanoma, providing a plausible mechanism for regrowth of melanoma at previous primary sites despite previous excision with histologically clear margins. It also explains why recurrences can arise after highly variable latent periods, ranging from months to up to 15 years, and at locations that are not immediately continuous with the primary excision scar. The extent of “field effect” is unrelated to thickness of the primary tumour, the parameter currently used to determine the width of safety margins in melanoma. In fact, according to the field cell concept, minimal residual disease is linked to the in situ portion, suggesting that the horizontal extent may be more important to estimate the risk of recurrence in acral melanoma (Bastian, 2003).

## 4 ALM BIOLOGY AND MICROENVIRONMENT

**FIGURE 2:**
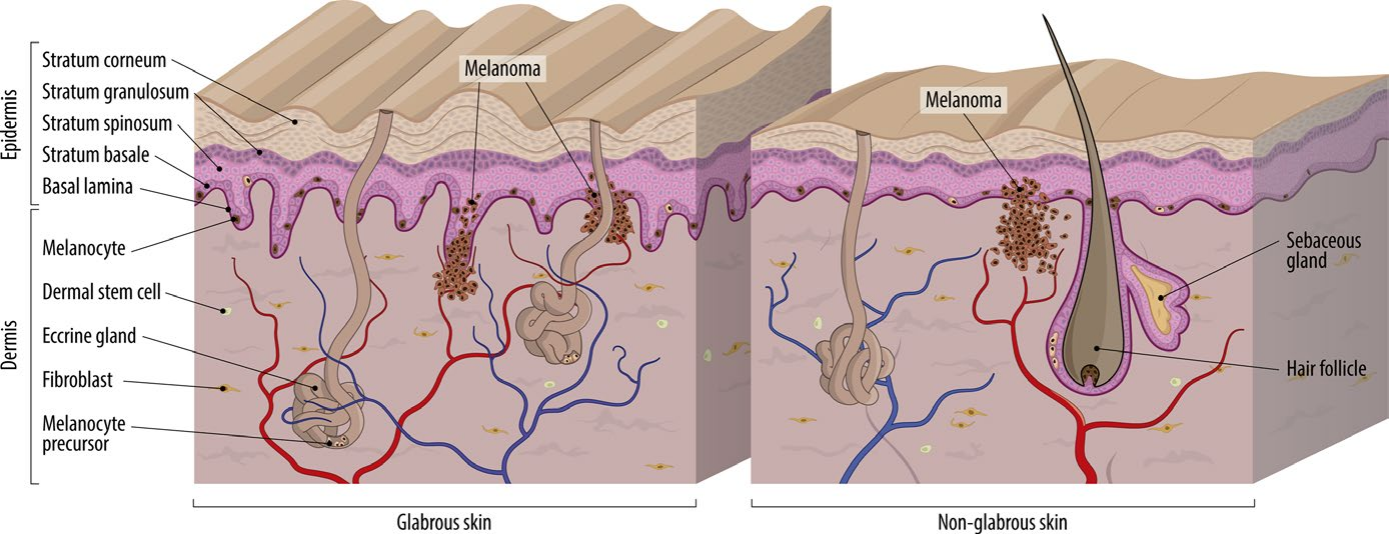
Schematic representation of glabrous and non‐glabrous skin. A thick stratum corneum, a grooved surface alternating ridges and sulci and the absence of hair follicles are some of the characteristics that define glabrous skin. Melanocytes are located in the basal layer of the epidermis of both skin types. Melanocyte precursors have been detected in hair follicles of non‐glabrous skin and in eccrine glands of glabrous skin. Evidence suggests that the epidermis may be a source of melanocyte precursors independent of skin appendages. Dermal stem cells can also differentiate into melanocytes. In glabrous skin, oncogenic mutations in melanocyte precursors have been observed in eccrine glands, suggesting that these can be transformed into melanoma cells. Other characteristics specific of glabrous skin such as the presence of encapsulated sense organs are not illustrated in the figure

Skin from acral sites is unique and shows striking differences from skin from other anatomical sites, including the absence of hair follicles and sebaceous glands, a compact, thick stratum corneum, hypopigmentation and the presence of encapsulated sense organs. Another peculiarity of acral skin is the presence of a grooved surface continuously alternating ridges and sulci, with eccrine ducts opening at the centre of the ridges (Calonje, Brenn, Lazar, & Mckee, 2011) (Figure 2).

The number of melanocytes in different areas of the body varies substantially (Szabo, 1954). In acral skin of palms and soles, between 40 and 270 melanocytes per 1‐mm length of the epidermal/dermal junction are observed. In the nail apparatus, this number is reduced to less than 20 melanocytes per 1 mm length of the epidermal/dermal junction (Fernandez‐Flores & Cassarino, 2017). Melanocytes in the acral skin are normally located in the two lowest layers of the epidermis, in single units, equidistant from each other.

The origin of adult melanocytes on acral skin appears to be different from that of melanocytes on non‐glabrous sites. All melanocyte precursors are derived from the neural crest, where melanoblasts originate and migrate to colonize the developing epidermis. In non‐glabrous skin, they are mainly detected in hair follicles, where they can differentiate into melanocytes or remain as melanocyte stem cells (Gola, Czajkowski, Bajek, Dura, & Drewa, 2012; Lang, Mascarenhas, & Shea, 2013). Differentiated melanocytes have also been identified in sebaceous glands, but whether these differentiate from resident melanoblasts or from other sources such as hair follicle precursors remains to be demonstrated (Jang, Kim, Lee, et al., 2014). In glabrous skin, melanocyte precursors have been detected in the secretory portion of eccrine glands, providing a source of differentiated melanocytes to the epidermis (Okamoto et al., 2014). Interestingly, melanoblasts harbouring *CCND1* amplification have been detected in the secretory portion of eccrine glands among human melanoma cells, suggesting that these cells could originate acral melanomas. *CCND1* encodes Cyclin D1, a protein that forms a complex with cyclin‐dependent kinase 4 (CDK4) or 6 (CDK6), and whose activity is required for G1/S transition. This cell distribution may explain the characteristic parallel ridge pattern of acral melanoma (Okamoto et al., 2014).

Epidermal appendages are not the only source of melanocytes. Glover and colleagues revealed the presence of distinct populations of melanocytes in the epidermis of mice lacking hair follicles and eccrine glands. These were characterized as pigment‐producing melanocytes with proliferative potential interspersed with a small percentage of amelanotic melanocytes, suggesting that these populations could serve as melanocyte reserves (Glover et al., 2015). Another alternative source of melanocytes already described in glabrous skin is the dermis. When embedded in the dermal compartment of skin reconstructs, low‐affinity nerve growth factor receptor (NGFR)‐positive dermal stem cells isolated from glabrous human foreskin differentiate into melanocytes and migrate from the dermis to the epidermis, aligning singly along the basal layer similar to what would be expected for melanocytes of non‐glabrous skin (Li et al., 2010). These results were confirmed by Kumar, Parsad, Rani, Bhardwaj, and Srivastav (2016), who isolated NGFR‐positive dermal stem cells from glabrous skin of vitiligo patients and demonstrated that these cells can be differentiated into melanocytes in vitro (Kumar et al., 2016). NGFR has been used as a marker for the isolation of neural crest stem cells, given that NGFR‐positive cells have self‐renewal capacity and display multipotent differentiation properties (Li et al., 2010). Whether this could also be a source of melanocytes of other anatomical locations remains to be addressed.

Apart from melanocyte differentiation, other major differences between acral and non‐acral skin relate to the cells and factors that constitute the tissue microenvironment. Skin from palms and soles, for instance, shows distinct gene expression profiles compared with non‐acral skin including enhanced expression of the genes encoding keratin 9, 6 and 16 (Bissonnette et al., 2016). Interestingly, keratin 9 is exclusively expressed in acral skin, and its downregulation leads to hyperpigmentation of plantar acral skin in mice, suggesting a role of the microenvironment in regulating melanogenesis and, perhaps, impacting melanocyte function and/or proliferation (Fu et al., 2014). A decrease in *CCL27* gene expression was also observed in acral skin (Bissonnette et al., 2016). CCL27 is a chemokine produced by keratinocytes that attracts T cells to the skin, where they play a role in inflammation (Homey et al., 2002).

Given the well‐studied role of the microenvironment in melanoma development, progression and therapy response, it is conceivable to speculate that melanomas arising in acral locations may be influenced by these microenvironment differences. But cancer development is also influenced by external environmental factors, which act differently according to the anatomical region. For instance, both trauma and UV can influence the structure and composition of the skin, including by generating an inflammatory environment (Liu, Zhang, Gao, & Li, 2016). Interestingly, cutaneous inflammation mediated by mast cells is sustained for longer in hairy skin compared with glabrous skin after chronic exposure to stress, illustrating how external factors may have different impacts depending on the type of skin (reviewed in Căruntu, Boda, Musat, Căruntu, & Mandache, 2014).

When looking at the characteristics of the immune infiltrate, the microenvironment can exhibit an immune desert (immunological ignorance), immune excluded or inflamed phenotype. Their cellular and molecular properties are determinants of melanoma progression and therapeutic response (Chen & Mellman, 2017). Differences in the presence of immune cells in ALM compared with other cutaneous melanoma subtypes have also been reported. It has been recently demonstrated that ALM presents increased numbers of M2 macrophages in both peritumoral and intratumoral areas compared with SSM and this was significantly associated with several histopathological characteristics predictive of adverse prognosis including tumour thickness, ulceration, mitotic rate and metastasis (Zúñiga‐Castillo, Pereira, & Sotto, 2018). Regarding tumour‐infiltrating lymphocytes (TILs), lower levels were associated with ALM compared with other cutaneous melanomas, as well as with higher Breslow, Clark levels and longer overall survival (Castaneda et al., 2017). In addition, an association between lower levels of the tumour‐suppressor protein, p16(INK4A) in tumour cells, and lower density of CD3+ and CD8+ TILs was found, suggesting a probable relationship between p16(INK4A) and TIL immune response in ALM (Castaneda et al., 2019). p16(INK4A) is involved in the tumour cell intrinsic regulation of cell cycle progression by blocking the activity of CDK4/6. Park and Kim (2017), however, found no association between the different cutaneous melanoma subtypes and the levels of TILs, but observed that high levels of peritumoral and intratumoral lymphocytes were associated with less recurrence and metastasis and with longer disease‐free survival only in ALM.

## 5 GENOMIC DRIVERS AND MUTATIONAL SIGNATURES

Elucidating the biological pathways involved in ALM pathogenesis is key to the development of new molecular‐based targeted therapies (Shim et al., 2017). Unfortunately, compared with CM, very few molecular studies have been performed on ALM tumours. Although limited, this body of literature already suggests compelling molecular differences between ALM and other melanoma subtypes.

### 5.1 Genomic drivers

**FIGURE 3:**
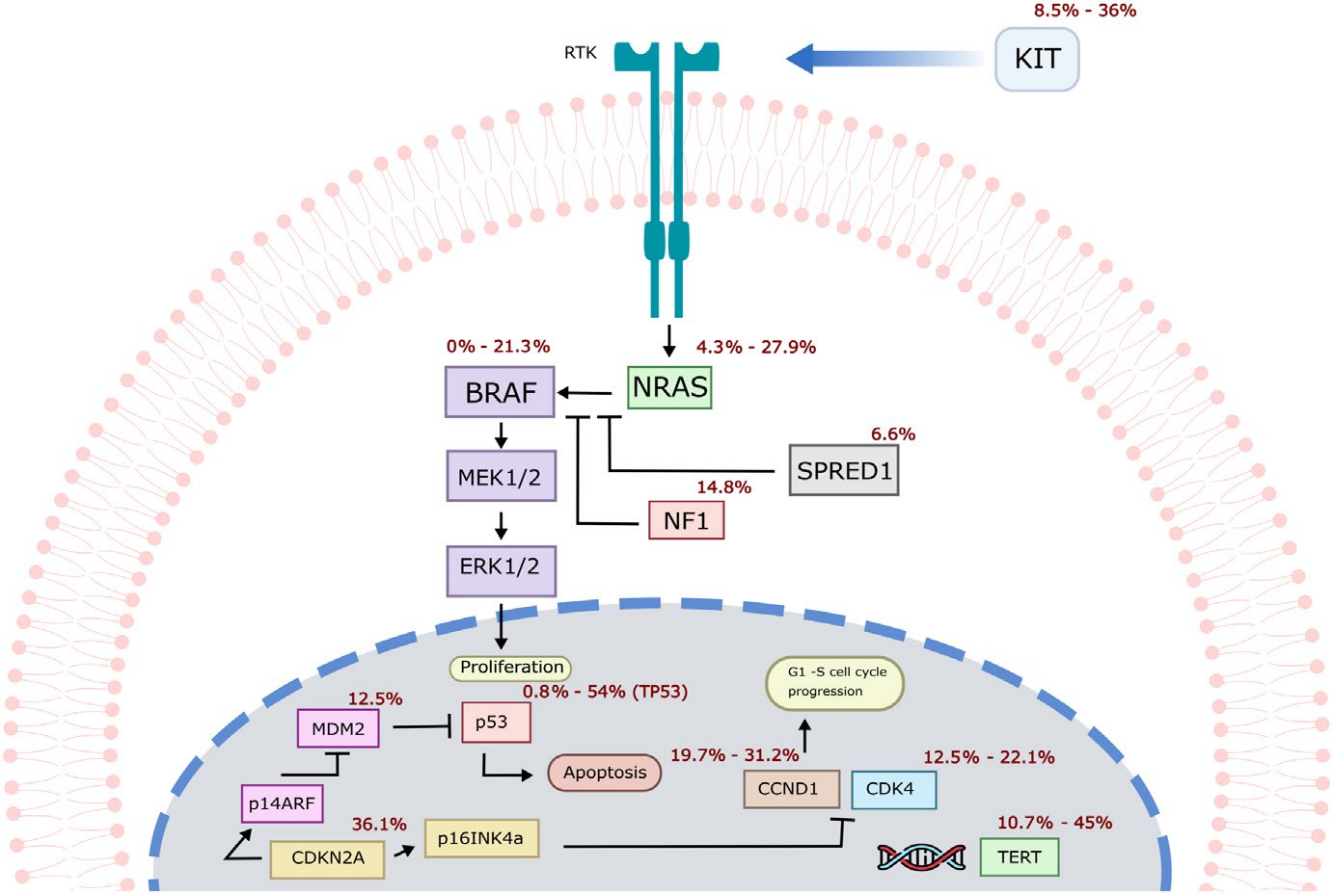
Representation of main pathways affected in acral lentiginous melanoma. Proportions of ALM cases that harbour alterations in the genes depicted are shown in red, based on the available data (Puig‐Butillé et al., 2013; Rabbie, Ferguson, Molina‐Aguilar, Adams, & Robles‐Espinoza, 2019; Shim et al., 2017; Yeh et al., 2019)

Sequencing and copy‐number profiling studies of ALM tumours have identified characteristic tumour‐promoting mutations implicating several genes. Examples of these include the KIT proto‐oncogene (*KIT*), which encodes a type III receptor tyrosine kinase that can stimulate cell growth, division, survival and migration; Cyclin D1 (*CCND1*) and cyclin‐dependent kinase 4 (*CDK4*), which encode a cyclin and a kinase that when bound to each other promote the transition from G1 to S phase; cyclin‐dependent kinase inhibitor 2A (*CDKN2A*), which encodes for two proteins: p16(INK4A) and p14(ARF), the latter of which helps stabilize TP53; telomerase (*TERT*)*,* encoding an enzyme crucial for telomere maintenance; and aurora kinase A (*AURKA*), which is involved in regulation of cell cycle progression by playing critical roles in mitosis (Curtin, Busam, Pinkel, & Bastian, 2006; Hayward et al., 2017; Puig‐Butillé et al., 2013; Yun et al., 2011). In this regard, a study over 514 ALM samples identified that these tumours frequently display a dysregulated CDK4 pathway, a product of either gains of *CDK4/CCND1* or loss of *CDKN2A* that, in turn, promote G1 to S cell cycle transition and thus contribute to tumour proliferation (Kong et al., 2017) (Figure 3). Further, as in other melanoma subtypes, *TERT* or its promoter are frequently mutated; for example, Liang et al. (2017) observed *TERT* aberrations in 41% of their patients with acral melanoma. In their study, Yeh et al. (2019) observed a low frequency of *TERT* promoter mutations but amplification of the *TERT* locus in 10.3% of the cases, and 3.3% cases with copy‐number transitions with relative gain of *TERT,* although the comprehensive role of telomere regulation specific to ALM has not been clarified (Hayward et al., 2017; Liang et al., 2017).

ALM stands out from other melanoma subtypes as it carries a large number of structural variants including duplications, deletions, translocations and inversions. Through whole‐genome sequencing of 35 ALM and 8 mucosal melanoma (MM) samples, Hayward et al. (2017) identified that, compared with any other melanoma subtype, both ALM and MMs carry significantly higher numbers of genomic rearrangements, in accordance with previous studies (Curtin et al., 2005). Furthermore, these alterations show evidence of being caused by repeated events of breakage–fusion‐bridge (BFB) and chromothripsis, and were found in ~68% of the acral samples studied. Such catastrophic events, although found in other cancer types, rarely reach the prevalence found in ALM, which highlights the uniqueness of the molecular aetiology of this disease (Rode, Maass, Willmund, Lichter, & Ernst, 2016).

Most studies of the molecular profile of ALM have been done in European‐descent populations, but there are a few from other populations available. A study from Shim et al. (2017) analysed the mutational profile of acral melanomas in a Korean population and reported mutational frequencies of 8.5%, 4.3% and 6.4% of *KIT*, NRAS proto‐oncogene (*NRAS*) and the BRAF proto‐oncogene (*BRAF*), respectively, and noted that their results were similar to those described in other Korean and Asian populations, but that mutation rates were lower than those observed in other studies. As *NRAS* and *BRAF* are among the most commonly mutated genes in other melanoma subtypes due to their crucial roles in triggering the mitogen‐activated protein kinase (MAPK) pathway, Shim et al. (2017) speculate that the differences between the mutation rates could reflect a different genetic and/or environmental cause between Asian and European populations.

Overall, the frequency of *RAS, BRAF* or neurofibromin 1 (*NF1*) mutations resulting in the activation of the MAPK pathway seems to be lower in ALM than in other subtypes of melanoma (Hayward et al., 2017; Vazquez et al., 2015; Yun et al., 2011), and other drivers such as *KIT*, *PAK1* and *SPRED1* may be more important (Curtin et al., 2006; Liang et al., 2017; Yeh et al., 2019) (Figure 3). *NF1* and *SPRED1* encode proteins involved in the regulation of the MAPK signalling pathway, while *PAK1* codes for a protein kinase involved in signalling pathways important for cell migration, proliferation, apoptosis and mitosis. However, a significant proportion of tumours still remain with no known drivers. These studies also suggest that chromosomal instability is a contributing factor to the aetiology of ALM and that it is different from that of melanomas in sun‐exposed skin.

### 5.2 Mutational signatures

The identification of mutational signatures from a collection of cancer genomes can pinpoint the processes, both environmental and endogenous, that were operative during tumour development and consequently that caused mutation accumulation. As expected, several mutational signature analyses on CM have identified a high prevalence of the UV‐induced signature dominated by C>T transitions (termed Signature 7) (Alexandrov et al., 2013). Unfortunately, few whole ALM genomes and exomes have been analysed in this way, as this subtype of melanoma is not well represented in some of the largest tumour collections such as The Cancer Genome Atlas (TCGA) (Cancer Genoma Atlas Network, 2015; Hayward et al., 2017), which only has 2 out of 470 samples classified as ALM. From the available analyses, however, we can gather that the dominant signature is not UV‐induced, as expected, but rather that two other signatures dominate the ALM mutational landscape, both of which are age‐related (Signatures 1 and 5) (Hayward et al., 2017).

Regarding structural rearrangements, a recent study that analysed 256 cutaneous melanomas observed that acral melanomas had an enrichment of a type of genomic rearrangement that the authors named “tyfonas” (Greek word meaning typhoon), which are extremely large amplicons comprising >100 Mb of genomic material and present in high copy number (often in more than 50 copies) (Hadi et al., 2019). The authors speculated that these rearrangements may be able to generate neoantigens, explaining the similar response rate to immune checkpoint inhibition therapy between acral and cutaneous melanoma (Shoushtari et al., 2016).

However, these conclusions were derived from a small number of tumours; therefore, our knowledge of the molecular events leading to ALM remains limited.

## 6 DISEASE MODELS AND POTENTIAL THERAPEUTIC TREATMENTS

Due to the poor prognosis associated with ALM, clinically relevant experimental models are urgently needed to identify biomarkers and drug targets. Unfortunately, these have not kept pace with those available for CM, for which there are several in vitro, in vivo and ex vivo models (Brohem et al., 2011; Krepler et al., 2017; Pérez‐Guijarro, Day, Merlino, & Zaidi, 2017; Smalley, Lioni, Noma, Haass, & Herlyn, 2008). As a consequence, little is known about the mechanisms of ALM development and there are no specific targeted therapeutic approaches available for the majority of *BRAF* wild‐type ALM patients.

There are no transgenic mouse models specific for ALM. In recent years, several melanoma patient‐derived xenograft (PDX) platforms have been developed, but few ALM specimens were included in the collections. Krepler et al. (2017) and Garman et al. (2017) together established 15 acral melanoma PDXs, which are included in a broad collection of 319 melanoma PDXs. Einarsdottir et al. (2014) established 23 melanoma PDXs, including one ALM PDX. A large melanoma PDX platform developed by Girotti et al. (2016) reported seven acral melanomas among a collection of 126 melanoma of other subtypes. Kong et al. (2017) established five ALM PDXs in a study exclusively focused on this subtype of melanoma. The generation of PDX platforms has had an important impact on the generation of acral melanoma cell lines.

Until 2012, only seven ALM cell lines derived from primary and metastatic lesions from different laboratories had been described (Ashida, Takata, Murata, Kido, & Saida, 2009; Furney et al., 2012; Murata et al., 2007; Satyamoorthy et al., 1997). More recently, additional cell lines have been reported, most of them derived from PDXs (Garman et al., 2017; Kong et al., 2017; Krepler et al., 2017; Liang et al., 2017). Fortunately, most of the ALM cell lines available have been, at least partially, genetically characterized. However, the number of available cell lines is still scarce and poorly captures the diversity of the disease. Scarcity of cell lines may be attributed not only to the rarity of this disease in Australia, Europe and North America, where most melanoma studies have been performed, but also to how difficult they are to establish. Acral melanoma cells depend on a specific cocktail of growth factors that differs from that of other cutaneous melanoma cells (Ashida et al., 2009; Furney et al., 2012; Murata et al., 2007; Satyamoorthy et al., 1997), but this formulation still needs to be refined. Of note, at least half of the acral melanoma cell lines developed to date are derived from primary tumours. This may also make the establishment of cell lines more difficult because these samples are often pigmented and contaminated with fungi. Moreover, primary tumours are highly dependent on the skin microenvironment and cell lines derived from those grow less aggressively in vitro (Satyamoorthy et al., 1997). This and other factors such as the low mutation burden of acral melanoma samples may have an impact on the low number of available cell line models.

Of major clinical importance, drug intervention experiments conducted with melanoma PDXs and derived cell lines clearly show that these models can recapitulate the therapeutic response observed in patients, can help identify the causal role of genetic alterations in therapy resistance and may be used to test drug combinations (Einarsdottir et al., 2014; Girotti et al., 2016; Kemper et al., 2016; Krepler et al., 2017). In the study by Liang et al. (2017), genetic alterations in *TERT* were found in more than 40% of acral melanomas in patients. *TERT* inhibition (by telomerase inhibitor IX) decreased cell viability of two acral melanoma cell lines with copy‐number gain and promoter mutations on *TERT*, whereas no effect was observed in normal melanocytes, suggesting the potential of *TERT* as a therapeutic target in ALM (Liang et al., 2017). In CM, it was recently shown that *TERT* inactivation significantly affects in vitro viability and in vivo tumour growth of melanoma cells, including cells and tumours resistant to targeted and immunotherapies (Zhang et al., 2018). The frequency of *TERT* alterations in ALM makes this melanoma subtype a candidate for this therapeutic approach.

Two previously described ALM cell lines harbour *KIT* mutations: SM3 (D820Y) and WM3211 (L576P) (Ashida et al., 2009; Furney et al., 2012). This is consistent with the presence of KIT alterations in ALM and other melanoma subtypes and has supported the development of studies exploring its role as a therapeutic target in melanoma (Curtin et al., 2006; Hodi et al., 2013). In ALM, KIT inhibition through dasatinib (but not other KIT inhibitors) decreased viability of WM3211 cells (Posch et al., 2016; Woodman et al., 2009). The potential clinical relevance of KIT as a therapeutic target was demonstrated by the significant reduction of tumour burden observed in patients treated with second‐line dasatinib (Woodman et al., 2009). Unfortunately, both patients eventually evolved to progressive disease, warranting further work exploring the role of KIT as a therapeutic target in ALM and other melanoma subtypes.

Oncogenic activation of the CDK4 pathway through copy‐number gains in *CDK4*, *CCND1* and loss of *CDKN2A* is a common genetic feature of acral melanomas (Bastian et al., 2000; Curtin et al., 2005; Furney et al., 2012; Kong et al., 2017; Liang et al., 2017). Kong et al. (2017) used ALM PDX with different CDK4 pathway aberrations to test response to either the pan‐CDK inhibitor AT7519 or the specific CDK4/6 inhibitor PD0332991 (palbociclib). While ALM PDXs with no alterations in the CDK4 pathway showed no significant response, tumour volume was decreased in PDXs harbouring *CDK4* gain/*CDKN2A* loss or *CCND1* gain/*CDKN2A* loss and, to a larger extent, in PDXs with copy‐number gains in both *CDK4* and *CCND1*. The effect on tumour growth correlated with decreased phosphorylation of RB Transcriptional Corepressor 1 (Rb1) (Kong et al., 2017). Complementary to these findings, Yu et al. (2019) showed that, in humanized mouse and PDX models, palbociclib improved response to immune checkpoint blockade. Although this was not observed specifically in acral melanoma models, supporting evidence suggests that these results may be applied to this melanoma subtype. Palbociclib treatment increased the expression of PD‐L1 and other genes involved in immune response, such as those related to the JAK/STAT pathway in acral melanoma cells harbouring *CDK4* gain/*CDKN2A* loss. Furthermore, activation of CDK4 pathway, in the case of acral melanoma patients achieved via *CCND1* copy‐number gains, was associated with response to PD‐1 blockade (Yu et al., 2019). These findings bring hope for the use of CDK4 inhibitors in a relevant clinical setting and may contribute to improve response to immunotherapies for acral melanoma patients. Clinical trials testing palbociclib in ALM patients with CDK4 pathway aberrations (NCT03454919) and testing checkpoint blockade specifically in acral lentiginous and mucosal melanoma patients (NCT02978443) are ongoing.

## 7 CONCLUSIONS

ALM is a complex disease that appears to have both weak germline genetic risk factors and an environmental component, potentially trauma associated with mechanical injury, involved in its aetiology. Germline components may have a role in cell cycle regulation, given the observation that these patients may have an increased risk of other types of cancer, or in melanocyte development, but larger analyses such as genome‐wide association studies are needed to establish whether there is a true genetic signal and what functional role it may have.

Diagnosis of ALM is difficult, being easily confused with other dermatological conditions, and because of this, it is usually diagnosed at later stages compared with other melanoma subtypes. However, whether this late diagnosis causes its poorer prognosis or whether ALM is inherently biologically more aggressive remains to be addressed. In this regard, dermoscopy is widely regarded as being the best method to diagnose promptly ALM, providing a considerable improvement in diagnostic accuracy over other examination methods.

Acral lentiginous melanoma also appears to be a different disease compared with CM, given the microenvironment differences between glabrous and non‐glabrous skin, the embryonic origin of acral melanocytes and its immune compartment. This may be reflected in the large differences in the genomic landscape of CM and ALM, with the latter having a much lower number of mutations but a higher number of large chromosomal aberrations. The mutational landscape is also strikingly different, with CM being dominated by a UV‐induced signature but ALM displaying age‐associated signatures. Definitely, large‐scale ALM studies are necessary for establishing genomic drivers and mutational signatures, and more functional analyses are needed to address possible aetiological factors.

Some ALM disease models are available, mainly in the form of PDX models and cell lines, but these are still scarce and have not been analysed in great depth. However, their study has already highlighted palbociclib as a possible agent in treating ALM patients with aberrations in the CDK4 pathway, which highlights the power of these kinds of preclinical studies and calls for more research for this subtype of melanoma. Given the strikingly low number of studies on this disease when compared to CM and that it is the most common form of the disease in some countries in Latin America, Africa and Asia, we truly believe it is about time we shift our focus to these regions of the world.

## CONFLICT OF INTEREST

None declared.
